# Genome-Wide Identification of the *APRR2* Gene Family and Rind Color Trait Analysis in Zucchini (*Cucurbita pepo*)

**DOI:** 10.3390/genes17091063

**Published:** 2026-09-01

**Authors:** Tongsheng Liu, Shuo Li, Ke Wu, Xinbin Wang, Xiaoyang Sun, Wenqi Ding

**Affiliations:** 1 College of Agriculture, Qingdao Hengxing University of Science and Technology, Qingdao 266100, China; 17662055965@163.com (T.L.);; 2 College of Grassland Science, Qingdao Agricultural University, Qingdao 266109, China; 18214491468@163.com (K.W.);

**Keywords:** *Cucurbita pepo*, *APRR2* gene family, rind color

## Abstract

Rind color is an important quality trait in zucchini (*Cucurbita pepo*). As a core transcription factor in plant pigment biosynthesis, *APRR2* plays a conserved yet mechanistically diverse regulatory role in the formation of rind color in various vegetables. However, the APRR2 transcription factor regulates rind color but has not been systematically identified in *C*. *pepo*. In this study, 50 *APRR2* genes were defined by the presence of the conserved REC domain verified. These genes were identified and found to be unevenly distributed across the 20 chromosomes, primarily expanded through tandem duplication events. Phylogenetic and structural analysis classified these genes into three distinct subgroups, all featuring the conserved REC domain essential for pigment regulation but exhibiting variations in motifs and intron–exon structures. Promoter analysis revealed abundant light-responsive, hormone-responsive and stress-responsive elements that may contribute to environmental adaptation and photomorphogenesis. Crucially, transcriptome analysis during rind development (0 and 10 days after pollination) in green (GR) and white (WR) rind lines demonstrated profound functional divergence. Expression clusters indicated temporal shifts in metabolism and enriched “circadian rhythm-plant” and “photosynthesis” pathways in WR at 0 DAP. Specific *APRR2* genes were tightly correlated with rind color. qPCR validation of the 24 selected *APRR2* genes classified them into four trend groups based on the direction of expression change at 10 DAP. In total, 14 genes were upregulated in both GR and WR, 6 were downregulated in both lines, 1 was upregulated in GR but downregulated in WR, and 3 were downregulated in GR but upregulated in WR. Furthermore, protein–protein interaction prediction and yeast two-hybrid (Y2H) assays detected a physical interaction in yeast between a core APRR2 protein and a bHLH62 transcription factor, suggesting a potential interaction that may be involved in rind color regulation, pending in planta validation. The study first identified the members of the *APRR2* gene family in *C*. *pepo* and conducted a bioinformatics analysis on them. The study establishes the molecular basis of APRR2 function and offers valuable resources for breeding improved *C*. *pepo* varieties.

## 1. Introduction

Cucurbita spp. is an economically important crop genus in the Cucurbitaceae family. It has five major species: *C*. *pepo*, *C*. *maxima*, *C*. *moschata*, *C*. *argyrosperma*, and *C*. *ficifolia* [1]. Zucchini, as a significant horticultural crop, is one of the top ten vegetables globally. China is the world’s dominant producer of *C*. *pepo*. In 2024, its total production reached approximately 28 million tons, representing 53% of the global total, and it has sustained its leading position over consecutive years (https://www.chinabmao.com/freereport/105538.html, accessed on 6 May 2025). Fruit rind color is a key trait determining external quality and maturity, affecting consumer preference and market value. The rind color of *C*. *pepo* exhibits a diverse range of colors, including light green, dark green, white, and yellow. Rind color is primarily determined by the accumulation and dynamic balance of three major pigments: chlorophylls, carotenoids, and anthocyanins [2]. These pigments collectively produce a diverse color range spanning green, yellow, orange, red, and purple. Hitherto, the metabolism regulation of these pigments involves complex molecular networks and multi-pathway interactions [3].

APRR2 (*Arabidopsis* pseudo-response regulator 2) belongs to the plant-specific family of pseudo-response regulators. Its typical structure comprises a pseudo-receiver domain, which is homologous to the response regulator domain of bacterial two-component systems. However, unlike typical response regulators, APRR2 in most angiosperms lacks the key phosphorylation-accepting site within this domain, hence it is termed “pseudo” [4]. Unlike typical regulators, most angiosperm APRR2s lack a key phospho-accepting residue in this domain. Studies in basal land plants reveal that its ancestral homologue *PpPRR2* retains a functional DDK motif capable of phosphotransfer in vitro [5]. As a core transcription factor in plant pigment biosynthesis, *APRR2* plays a conserved yet mechanistically diverse regulatory role in the formation of rind color in various vegetables. In tomato (*Solanum lycopersicum*), the *APRR2* gene was first identified as being associated with fruit pigment accumulation using artificial neural network analysis. Its expression was significantly upregulated during the breaker stage, and its overexpression increased the number and size of plastids, consequently increasing chlorophyll content [6]. Genome-wide association studies in eggplant (*Solanum melongena*) germplasm resources pinpointed SmAPRR2 as the major gene responsible for chlorophyll accumulation in the fruit rind, with multiple loss-of-function mutations confirmed as key factors leading to the white peel phenotype [7]. In watermelon (*Citrullus lanatus*), *ClAPRR2* was mapped as a key gene regulating light-colored stripes on the fruit rind, primarily determining the stripe phenotype by affecting chloroplast development and chlorophyll metabolism. In light-green striped materials, a 12-bp insertion in this gene causes premature translation termination and altered subcellular localization [8]. In pepper (*Capsicum annuum*), a dCAPS marker developed based on mutation sites in the CaAPRR2-like gene can effectively distinguish between creamy white and green fruits [9]. In *C*. *pepo*, a 2.2-kb 5′ deletion in CpAPRR2 underlies induced white compared to dark green skin [1]. Thus, APRR2 acts as a central positive regulator of pigmentation in these crops, though regulatory mechanisms diverge. These include transcriptional activation (tomato), loss-of-function via structural variation (eggplant, summer squash), protein alteration via coding insertion (watermelon), and multigenic coordination expression (pepper).

Previous studies show that APRR2 transcription factors serve as pivotal regulators of pigment accumulation in plant organs including fruits and roots, with their functional outcomes strictly determined by selective protein interactions. In cucumber (*Cucumis sativus*), CsAPRR2 forms a transcriptional complex with CsTCP15 and CsTCP20B to regulate the expression of genes such as photosystem subunits, influencing chloroplast development through sulfur metabolism regulation [10,11]. Strikingly contrasting in carrot (*Daucus carota*), DcAPRR2 functions as a core activator for carotenoid biosynthesis but is directly inhibited on binding by the suppressor protein DcRPGE1. Within white carrot roots, functional DcRPGE1 binds and sequesters DcAPRR2, preventing its binding to promoters of carotenoid synthase genes (DcPSY1, DcPSY2, DcLCYE) and thereby blocking pigment production. Conversely, orange and yellow carrot roots harbor mutated, dysfunctional DcRPGE1 that lacks interaction capacity with DcAPRR2. Consequently, liberated DcAPRR2 binds its target promoters, activating the carotenoid pathway and enabling root pigment accumulation [12]. In tomato, a regulatory paradigm exists in which the chlorophyll-associated gene SlAPRR2-like1 itself becomes a target. Its promoter is bound by a transcription-repressing complex consisting of SlKN5 and SlBLH proteins. This complex binding suppresses SlAPRR2-like1 expression, which in turn simultaneously represses chlorophyll synthesis and promotes carotenoid accumulation, ultimately driving the critical green to red color shift during the fruit breaker stage [13]. Therefore, APRR2 acts as a central regulatory hub by assembling into profoundly different protein complexes. However, the underlying mechanisms governing this process in *C*. *pepo* are currently unknown and warrant systematic elucidation.

High-quality complete genomes are essential for genetics and genomics research. The first genomes of *C*. *maxima* and *C*. *moschata* were constructed using short-read sequencing and released in 2017, followed by the assembly of the *C*. *argyrosperma* genome in 2021 [14]. In 2024, the *C*. *maxima* genome was re-sequenced, resulting in the first telomere-to-telomere (T2T) gap-free complete pumpkin genome, which was used to analyze the unique centromere structure and characteristics in pumpkins [15]. Genome sequencing has facilitated the investigation of key evolutionary events in Cucurbitales and the timing of introgression of domestication genes (*C*. *argyrosperma*) [16]. However, progress on the *C*. *pepo* genome has been relatively slow, and gene family analyses have also advanced at a limited pace. While gene families such as *JAZ* and *PLATZ* genes have been identified, the *APRR2* gene family has not yet been characterized in *C*. *pepo* [17,18]. Therefore, this study will conduct the screening, identification, sequence alignment, and phylogenetic classification of *APRR2* gene family members in *C*. *pepo* using its genomic data. Analyses of protein conserved domains and motifs, as well as predictions of protein physicochemical properties and subcellular localization, will be performed. Furthermore, intra- and inter-family collinearity analyses of the *APRR2* gene family will be carried out. Transcriptomic analysis and expression profiling of the *APRR2* gene family will be performed during the color transition stage in two *C*. *pepo* accessions: green rind (GR) and white rind (WR). Subsequently, the candidate APRR2-interacting proteins (bHLH62) were predicted using bioinformatics methods and tested using yeast two-hybrid experiments. Based on the above analyses, this study aims to provide a theoretical foundation for untangling the biological functions of *APRR2* genes in the formation of *C*. *pepo* and to supply key genetic resources and molecular markers for breeding.

## 2. Materials and Methods

### 2.1. Plant Materials and Growth Conditions

The study employed two highly inbred C. pepo varieties: green rind (GR) and white rind (WR). GR served as the recurrent parent in a backcrossing scheme to the BC_6_F_1_ generation. GR is a high inbred line of *C*. *pepo* from Neimenggu 122 region, and WR is a high inbred line isolated from *C*. *pepo* cultivar Ruifeng. Subsequent self-crossing yielded the BC_6_F_2_ population. From this population, co-segregating markers within the target interval enabled the selection of homozygous GR and WR plant lines, thereby establishing transcriptome-sequencing near-isogenic lines (NILs) [19]. All plant materials were maintained by the Pumpkin Breeding Laboratory, College of Horticulture and Landscape Architecture, Northeast Agricultural University. NIL plants were grown in a greenhouse using standard ridge planting (50 cm plant spacing, 140 cm row spacing, 10 plants per ridge), with a 16-h photoperiod under 2000 μmol·m^−2^·s^−1^ and a relative humidity of at 60 ± 5%. Vines were vertically trellised when plant height reached ~30 cm. Standard horticultural practices including irrigation, hand pollination, and hand weeding were applied [19]. Plant rind epidermal tissue was collected at two developmental stages: 0 days after pollination (DAP) and 10 DAP. Samples comprised GR0 (green rind, 0 DAP), WR0 (white rind, 0 DAP), GR10 (green rind, 10 DAP), and WR10 (white rind, 10 DAP). Each sample included three biological replicates, totaling 12 samples.

### 2.2. Identification of APRR2 Family Members

The whole-genome sequence and annotation file for *C*. *pepo* were downloaded from the Cucurbit Genomics Database (ftp://cucurbitgenomics.org/pub/cucurbit/genome/C. pepo/, accessed on 6 January 2026). Protein sequences of nine *A*. *thaliana APRR2* genes containing conserved REC domain were retrieved from TAIR (https://www.arabidopsis.org/, accessed on 7 January 2026). We used these nine *A*. *thaliana* sequences as queries to perform a local BLAST search against the *C*. *pepo* genome annotation. Redundant hits were removed to obtain candidate *APRR2* sequences. These candidate sequences were subsequently validated using the InterPro database (https://www.ebi.ac.uk/interpro/, accessed on 8 April 2026) and NCBI BLASTp (https://www.ncbi.nlm.nih.gov/, accessed on 9 April 2026). The final verified sequences were designated as members of the *APRR2* gene family in *C*. *pepo*.

### 2.3. Multiple Sequence Alignment and Phylogenetic Analysis

Multiple sequence alignment of the full-length 50 APRR2 proteins from *C*. *pepo* and 9 from *A*. *thaliana* was performed using the Align Protein feature in MEGA 12 software. Following alignment, a phylogenetic tree was constructed using the neighbor-joining (NJ) method applying the Poisson correction model, utilizing pairwise deletion, and incorporating bootstrap analysis with 1000 replicates [20]. The resulting phylogenetic tree was visualized as a circular layout using the iTOL website platform (https://itol.embl.de/, accessed on 9 May 2026). Based on the topology of this circular tree, *APRR2* genes were classified into clades. Genes supported by a bootstrap value ≥ 70 were assigned to specific clades, whereas those with values below 70 were not assigned to any specific group [21].

### 2.4. Analysis of Gene Structure and Conserved Motifs

Conserved domain analysis of the APRR2 protein sequences was performed using the NCBI Conserved Domain Database (CDD), following sequence submission. Gene structures were visualized with Tbtools (v2.056). Separately, conserved motifs were identified using the MEME Suite and visualized using Tbtools [22].

### 2.5. Protein Physicochemical Properties and Subcellular Localization Prediction

The amino acid count, molecular weight, isoelectric point (pI), instability index, aliphatic index, and grand average of hydropathicity (GRAVY) of the 50 APRR2 proteins were analyzed using the ProtParam tool on the Expasy website (https://www.expasy.org/, accessed on 9 May 2026). Subcellular localization of these proteins was predicted using WoLF PSORT (https://wolfpsort.hgc.jp/, accessed on 9 May 2026).

### 2.6. Promoter Sequence Analysis

Putative promoter regions, defined as the 2000 bp sequence upstream of the start codon for each *C*. *pepo APRR2* genes, were retrieved from the genome assembly and annotation files using the Gtf/Gff Sequences Extract tool within Tbtools. These promoter sequences were subsequently extracted using the Fasta Extract tool in Tbtools, and their lengths were validated as 2000 bp using Fasta stats. The confirmed promoter sequences, with defined coordinates, were submitted to the PlantCARE database (https://bioinformatics.psb.ugent.be/webtools/plantcare/html/, accessed on 6 January 2026) for cis-regulatory element prediction. The resulting PlantCARE outputs were visualized using the simple bioSequence viewer tool [23].

### 2.7. Chromosomal Localization and Covariance Analysis of APRR2

Chromosomal locations of all *APRR2* genes were determined from the *C*. *pepo* genome annotation using the “gene location visualize from GTF/GFF” in Tbtools. Intraspecific collinearity relationships among *APRR2* genes of *C*. *pepo* were identified using the one step MCScanX tool in Tbtools (E-value < 1^−10^, number of hits ≤ 5). This analysis enabled visualization of APRR2 chromosomal positions, predicted tandem duplicates, and segmental duplications using the Advanced Circle visualizer in Tbtools. We then retrieved putative homologous APRR2 protein sequences in cucumber (*C. sativus*) and melon (*C. melo*) from the cucurbit genomics database (CuGenDB; http://cucurbitgenomics.org/). Interspecific syntenic relationships were analyzed separately between *C*. *pepo* and cucumber as well as *C*. *pepo* and melon using the one step MCScanX tool (identical parameters) and visualized using the multiple synteny plot tool.

### 2.8. Transcriptome Analysis

Total RNA was isolated from *C*. *pepo* rind epidermal tissue at designated developmental stages using TRIzol (RNAiso Plus; Takara, Japan). RNA quality was assessed using an SMA3000 UV spectrophotometer. Qualified samples were submitted to Novogene Co., Ltd. (Beijing, China) for transcriptome sequencing and analysis. Libraries were constructed and paired-end (150 bp) sequencing was performed on an Illumina HiSeq 4000 platform following the manufacturer’s protocol. Raw reads were processed with fastp software (version not specified, https://github.com/opengene/fastp, accessed on 6 January 2026) to remove adapters and low-quality sequences. Subsequent analyses utilized only these high-quality clean reads.

Quality-controlled reads were aligned to the *C. pepo* reference genome using HISAT2 v2.0.5. Genome-guided transcript assembly was performed for all samples using StringTie v1.3.3b. Transcript abundance was quantified as fragments per kilobase of transcript per million mapped reads (FPKM). Genes with FPKM exceeding 1 in at least one sample were characterized as expressed genes for further analysis. K-means cluster analysis of highly ex-pressed genes was performed using the R package clusterGVis (vO.0.8) (https://github.com/junjunlab/ClusterGVis, accessed on 6 January 2026) with the default parameters. The selected genes were subjected to KEGG functional enrichment analysis, and five pathways were chosen for mapping (*p*-value < 0.05). The DEGs were screened using R package DEseq2 (v1.44) by applying the thresholds of log2 (foldchange) > 1 and adjusted *p*-value < 0.05.

### 2.9. Yeast Two-Hybrid Assays

The amino acid sequence encoded by CpAPRR2 (Cp4.1LG05g02070.1) was imported into STRING (https://cn.string-db.org/) to analyze and predict proteins that may interact with CpAPRR2. The coding sequence of CpAPRR2 and CpbHLH (Cp4.1LG012g10810.1) were isolated from the cDNA of *C*. *pepo*. Using the specific primers listed in Supplementary Table S1, the coding sequences (CDS) of CpAPRR2 and CpbHLH were cloned into the pGBKT7 vector and transformed into the yeast strain Y2HGold according to the manufacturer’s instructions (Angyu, Shanghai, China).

The yeast strain Y2H harboring pGBKT7-CpAPRR2 + pGADT7-CpbHLH was serially diluted and plated on SD-Trp/-Leu medium. PGBKT7-53 + pGADT7-T served as the positive control, and pGBKT7-lam + pGADT7-T served as the negative control. The plates were incubated at 28 °C for 3–5 days.

Three larger colonies were selected, resuspended in 0.9% NaCl, and diluted to 10X, 100X, and 1000X. The yeast strain Y2H harboring pGBKT7-CpAPRR2 + pGADT7-CpbHLH was plated on SD-Trp/-Leu/-Ade/-His/X-α-Gal medium. A 2 μL aliquot of each dilution was spotted onto SD/-Trp/-Leu/-Ade/-His/X-α-Gal agar plates and incubated at 30 °C for 5 days, with X-α-Gal at a final concentration of 40 μg/mL. After incubation at 28 °C for 5–7 days, colonies were analyzed for the presence of pGBKT7-CpAPRR2 + pGADT7-CpbHLH at all dilution concentrations.

### 2.10. qRT-PCR Analysis

Total RNA was extracted from flesh tissue of DR and WR using the Quick RNA Isolation Kit (Huayueyang, Beijing, China), with three biological replicates per sample. First-strand cDNA was synthesized using the MonScript™ RTIII All-in-One Mix with dsDNase (Monad Biotech, Suzhou, China). qRT-PCR was performed on a Bio-Rad CFX96 Touch™ instrument using Taq Pro Universal SYBR qPCR Master Mix (Vazyme, Nanjing, China), with *Cpaction* as the internal reference gene, and gene-specific primers are listed in Table 1. Each reaction was run in triplicate. Relative expression levels were calculated using the 2^−ΔΔct^ method. The primer sequences of 24 *APRR2* genes and *Cpaction* sequences are listed in Supplementary Table S2.

### 2.11. Data Analysis

Graphs were plotted using R version 4.5.3 (www.r-project.org) and GraphPad Prism v9.00 (Graphpad Company, Boston, MA, USA). Heatmaps were generated using Tbtools software, and figures were refined using Adobe Illustrator software (v 30.0).

## 3. Results

### 3.1. Identification and Characterization of APRR2 Family Members in C. pepo

The genome and gene annotation files of *C*. *pepo* were obtained from the reference genome. A total of 50 members in *C*. *pepo* were identified with BLAST alignment screening using nine APRR members from *A*. *thaliana* and two cloned genes (Cp4.1LG05g02070.1, Cp4.1LG05g02060.1). This was followed by protein alignment verification on the NCBI website. The lengths of the APRR2 proteins ranged from 201 to 692 aa, with molecular weights between 21.58 kDa (Cp4.1LG10g02760.1) and 77.09 kDa (Cp4.1LG03g11620.1), indicating significant structural size variation within this family. The isoelectric point (pI) values ranged from 4.96 to 9.44 (e.g., pI = 4.96 for Cp4.1LG05g02060.1 and pI = 9.44 for Cp4.1LG10g02760.1). The large pI span (>4.48 units) suggests potential functional diversity in different cellular pH microenvironments. The instability index ranged from 32.78 to 78.88 (e.g., lowest = 32.78 for Cp4.1LG03g09290.1, highest = 78.88 for Cp4.1LG02g02600.1). With 88% of members (44/50) having an instability index > 40 (the classification threshold), they were classified as unstable proteins. The GRAVY values were all negative (−0.969 to −0.347). For instance, Cp4.1LG03g09110.1 exhibited the strongest hydrophilicity (−0.969), while Cp4.1LG08g02780.1 showed the weakest hydrophilicity, confirming that all members are hydrophilic proteins. All 50 APRR2 proteins were predicted to be localized in the nucleus, which is fully consistent with the characteristic nuclear localization and transcriptional regulatory function of the APRR2 family (Table 1).

### 3.2. Phylogenetic and Gene Structure Characterization of APRR2

**Figure 1:**
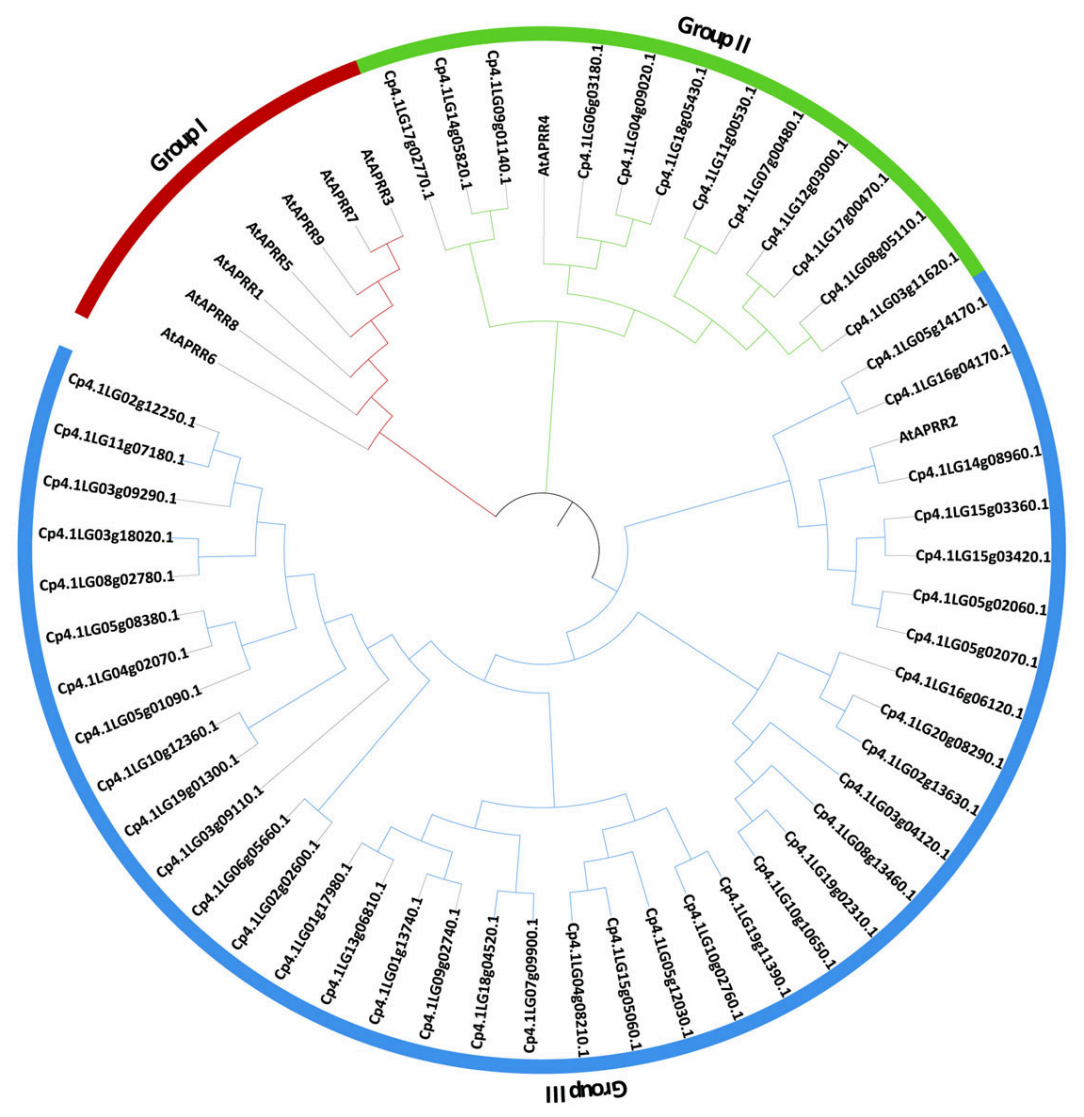
Phylogenetic analysis of *APRR2* family in *C*. *pepo* and *A*. *thaliana*, classifying 59 APRR2 into three subgroups: Group I, Group II and Group III. Cp = *C. pepo*; At = *Arabidopsis thaliana*.

Based on amino acid sequences of APRR2 family members from *C*. *pepo* and *A*. *thaliana*, phylogenetic analysis using MEGA 12 classified 59 members into three distinct subgroups (Figure 1). Group I, the smallest clade (7 members), comprised exclusively *A*. *thaliana* APRR proteins and formed an evolutionary distinct lineage. In contrast, Group III represented the largest subgroup (39 members), dominated by *C. pepo* APRR2 proteins (38 members) alongside a single *A*. *thaliana* APRR2 ortholog. Group II contained 13 members, including one *A*. *thaliana* APRR4 and twelve *C*. *pepo* APRR2 homologs. These divergence patterns indicate differential evolutionary trajectories and potential functional diversification of the APRR2 family between the species.

**Figure 2:**
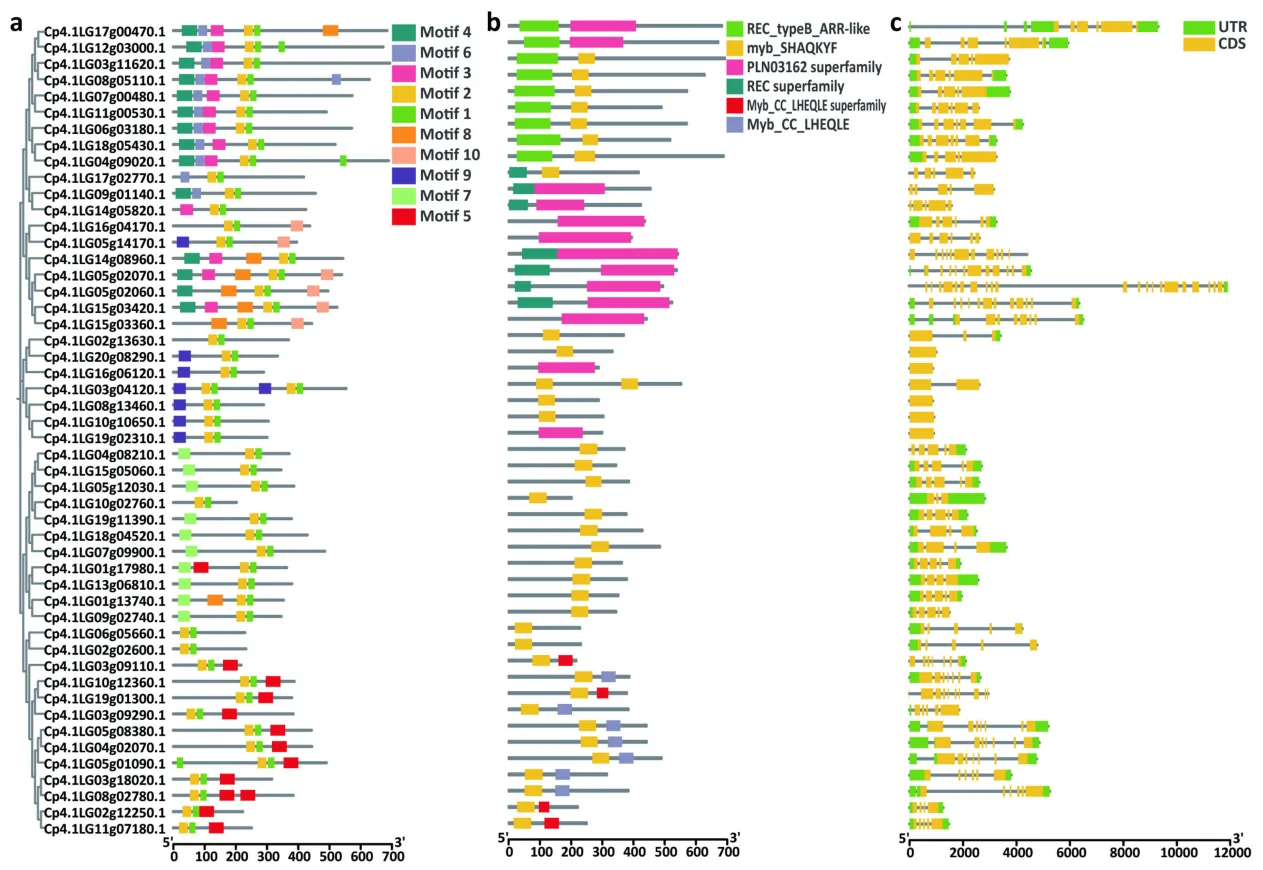
Analysis of gene structure, motif, and domains of CpAPRR2s. The phylogenetic tree based on the CpAPRR2 sequences. (**a**) Conserved motifs of CpAPRR2s. Different colored boxes display different motifs. (**b**) The domains of CpAPRR2s. Different colored boxes display different domains. (**c**) The exon–intron structure of CpAPRR2s. Green boxes represent UTR regions, yellow boxes represent exons, and introns are marked with black horizontal lines.

Conserved protein motifs and domains of the *APRR2* family were analyzed using MEME and NCBI Conserved Domains. Ten conserved motifs (motifs 1–10) were identified across all members. Motifs 1 and 2 were universally conserved in all subgroups. Structural conservation extended to domains within subgroups, although exceptions occurred. Motifs 4 and 6 were absent only in Cp4.1LG17g02770.1 and Cp4.1LG14g05820.1 of Group II (Figure 2a). All 50 *C*. *pepo* APRR2 proteins contained the core REC and Mfs_CC_UNKEGUE superfamily domains characteristic of plant *APRR2s*. Some additionally harbored auxiliary domains (e.g., PUG03162 superfamily, Myb-like/SANT domain). Domain analysis revealed differential conservation. Group II members retained the most complete core domain architecture, whereas Groups I and III displayed domain reduction or simplification (Figure 2b). Gene structure analysis showed substantial variation in UTR/CDS lengths and intron–exon organization. Exon numbers ranged from 1 to 22 (introns: 0–5). Group I contained 5–7 exons (0–5 introns), while Group III exhibited broad structural diversity (1–22 exons; 0–4 introns). These intron–exon patterns correlated with the observed motif and domain differentiation across subgroups (Figure 2c).

### 3.3. Analysis of Cis-Acting Elements in the Promoters of APRR2

**Figure 3:**
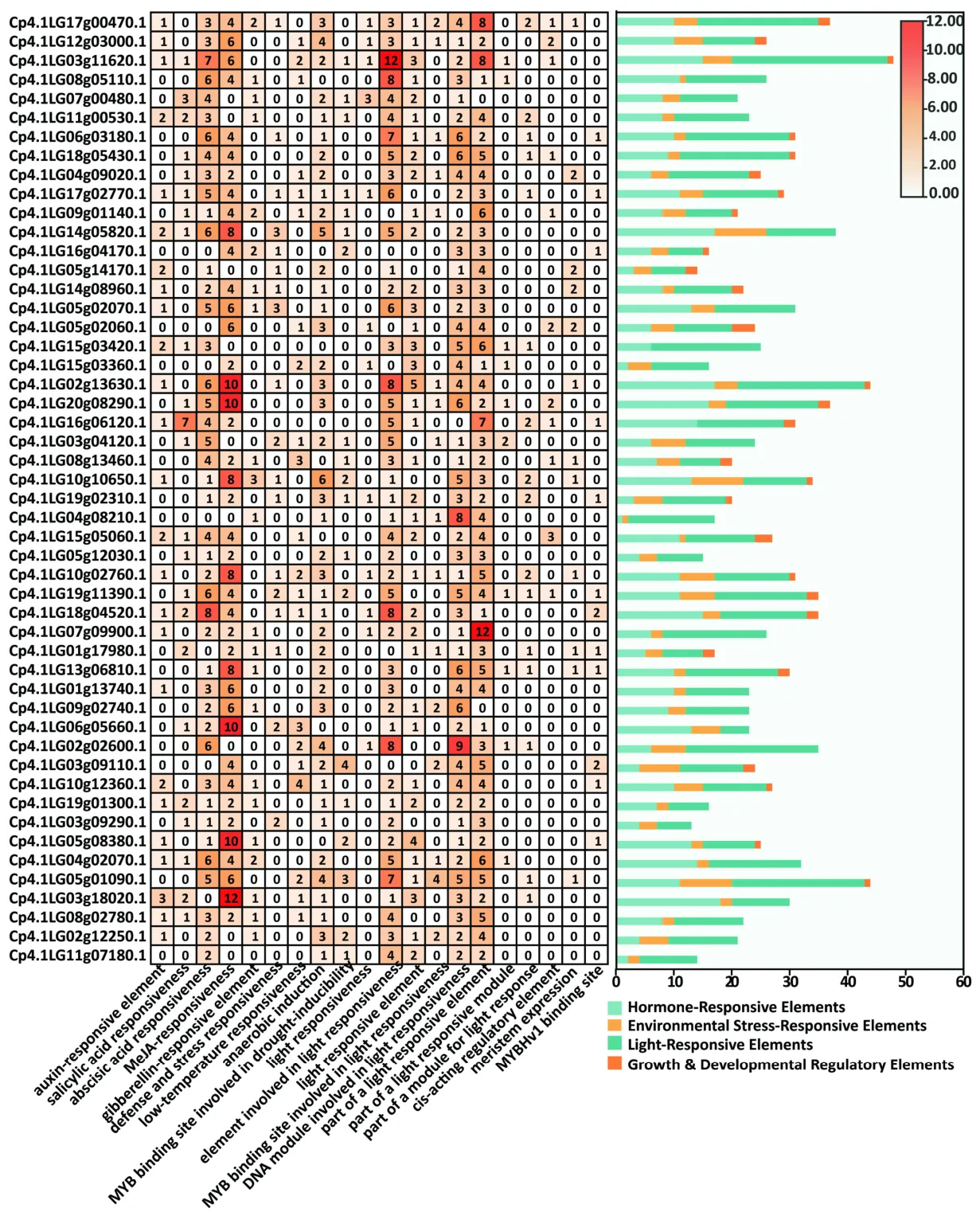
The distribution of cis-acting elements in the promoters of CpAPRR2s in *C*. *pepo*. The left heatmap illustrates the distribution and abundance of cis-acting elements in the promoter regions of each gene. On the right, the quantities of each type of component are listed. Rows correspond to gene IDs, and columns represent functional classifications of the elements.

To further investigate the functions of the *APRR2* gene family, we extracted 2000 bp upstream sequences for promoter cis-acting element analysis. Results revealed that multiple *APRR2* genes share common functional elements. For instance, both Cp4.1LG03g11620.1 and Cp4.1LG08g05110.1 contain light-responsive and abscisic acid-responsive cis-acting elements. Based on functional characteristics, the identified cis-acting elements were broadly categorized into four classes: hormone-responsive elements (e.g., auxin-responsive elements), environmental stress-responsive elements (e.g., low-temperature-responsive elements), light-responsive elements, and growth regulatory elements (e.g., meristem expression-related elements). Among these, light-responsive elements were the most abundant, comprising eight distinct types. Additionally, the distribution of cis-acting elements varied among the genes. For example, Cp4.1LG02g12250.1 and Cp4.1LG11g07180.1 lacked low-temperature-responsive elements, suggesting potentially reduced sensitivity to low-temperature environments (Figure 3).

### 3.4. Chromosomal Localization and Collinearity Analysis of APRR2

**Figure 4:**
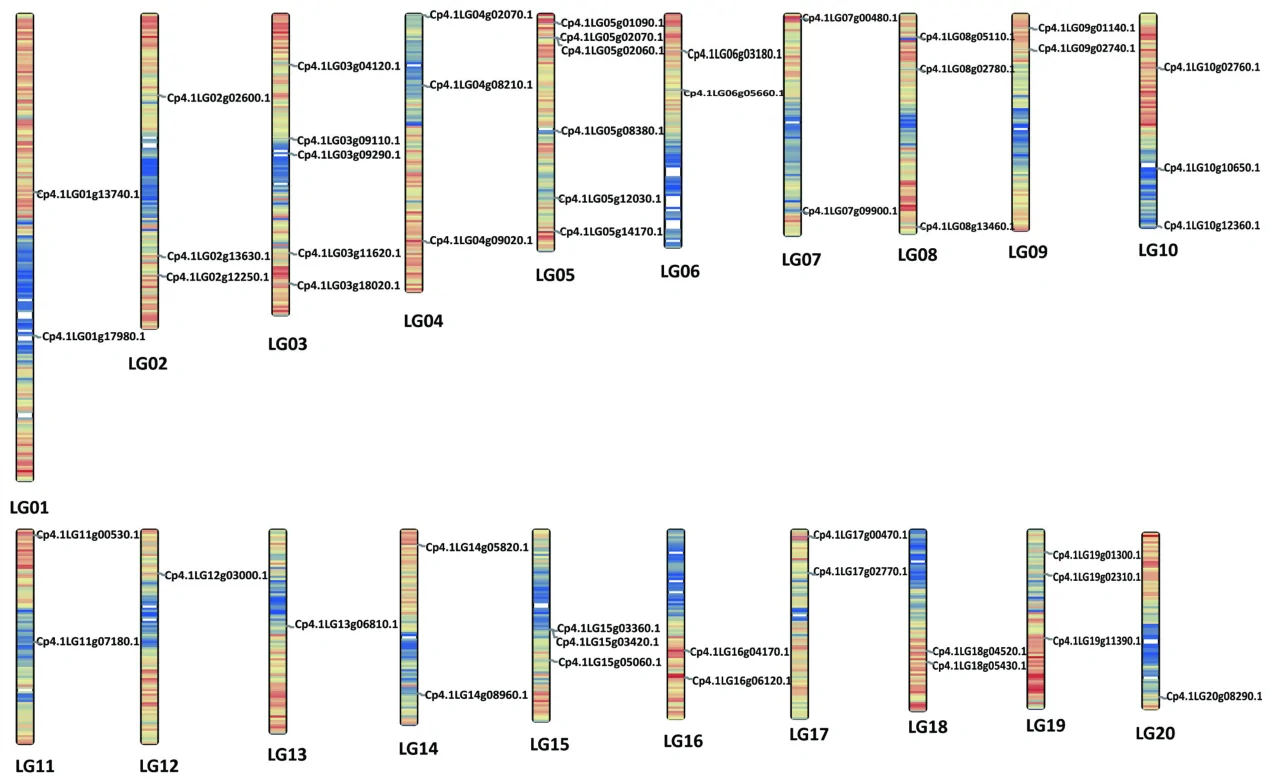
The 50 CpAPRR2s were located on the chromosomes. The length of each chromosome is shown in Mb, the chromosomal number is shown below the chromosome, and the *CpAPRR2s* are located on the right side of the chromosome.

The 50 *APRR2* genes were unevenly distributed across the 20 chromosomes of *C*. *pepo*. Chromosome Chr05 contained the highest number (six genes), while chromosomes Chr12, Chr13, and Chr20 each possessed only a single gene. Chromosomes containing two *APRR2* genes were Chr01, Chr06, Chr07, Chr09, Chr11, Chr14, Chr16, Chr17, and Chr18. Chromosomes with three genes included Chr02, Chr04, Chr08, Chr10, Chr15, and Chr19, and chromosome Chr03 contained five *APRR2* genes (Figure 4).

**Figure 5:**
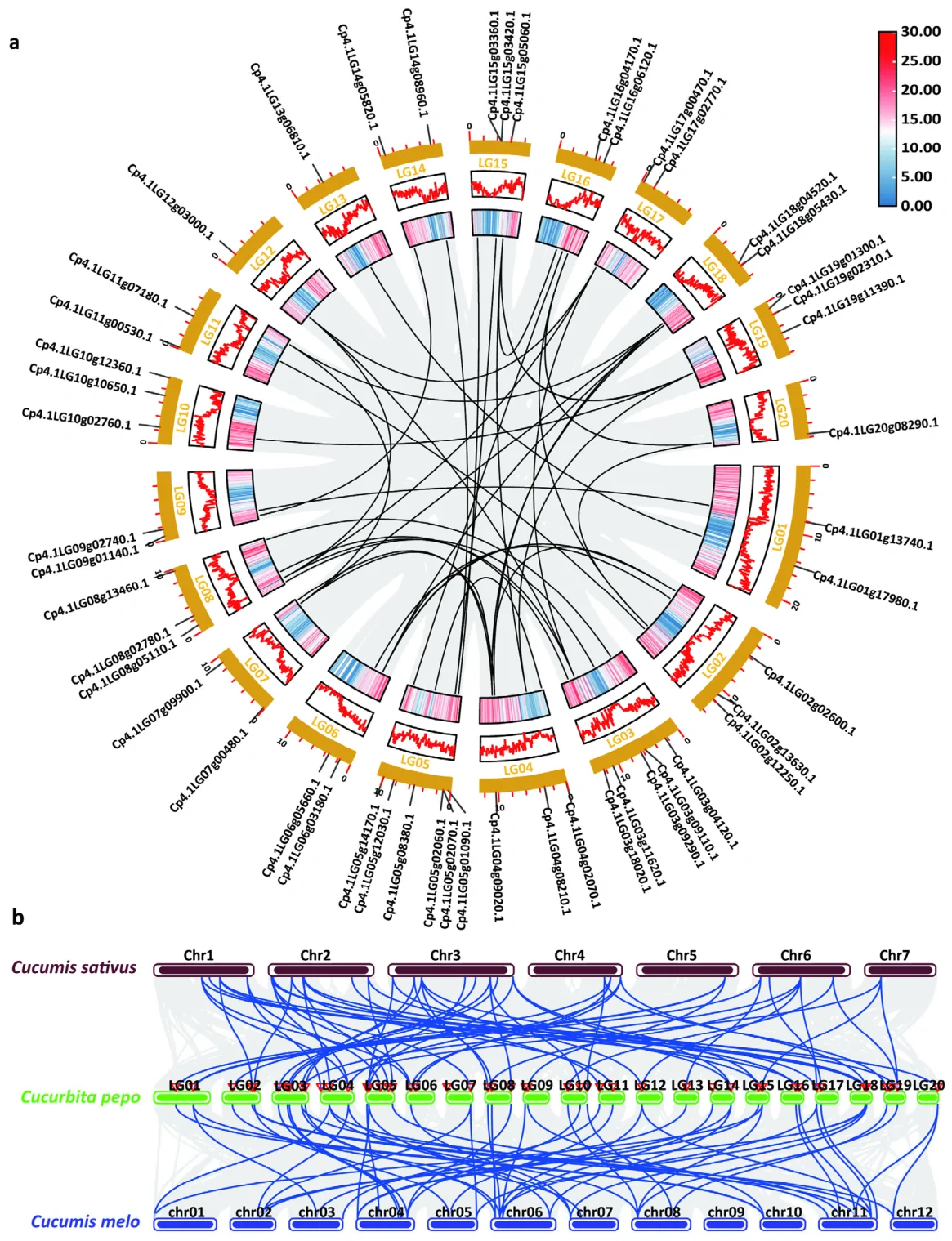
Circular representations of CpAPRR2 chromosomal dispersal and inter-chromosomal interactions, as well as synteny analysis of CpAPRR2 genes in melon (*C*. *melo*) and cucumber (*C*. *sativus*). (**a**) Analysis of syntenic relationships between CpAPRR2 paralog pairs; within the schematic image, duplicate pairs of CpAPRR2s are shown by black lines. (**b**) Analysis of syntenic relationships in *C*. *melo*, *C*. *melo*, and *C*. *pepo*, as shown by blue lines.

Intraspecies collinearity analysis of *C*. *pepo APRR2* genes using BLAST (2.14.0) and MCScanX (v1.0.0) revealed evidence of whole-genome duplication events, specifically identifying two tandem duplication events: one pair on Chr05 (Cp4.1LG05g02060.1::Cp4.1LG05g02070.1) and one pair on Chr15 (Cp4.1LG15g03360.1::Cp4.1LG15g03420.1) (Figure 5a). Consistent with their low gene count, Chr12, Chr13, and Chr20 were relatively gene scarce, with each harboring only a single *APRR2* gene. To explore the evolutionary mechanisms of the *APRR2* family, we also conducted interspecies collinearity analysis among *C*. *pepo*, *C*. *melo*, and *C*. *sativus*. This identified 61 orthologous gene pairs between *C*. *pepo* and *C*. *sativus* and 57 orthologous gene pairs between *C*. *pepo* and *C*. *melo*. The findings suggest that *C*. *pepo APRR2* genes likely share similar functions and originated from common ancestral genes. Further analysis revealed no significant correlation between gene linkage and either the number of *APRR2* genes or gene length on the chromosomes. Furthermore, genes exhibiting linkage were found to be positioned closely together on the phylogenetic tree (Figure 5b).

### 3.5. Transcriptome Data Analysis

**Figure 6:**
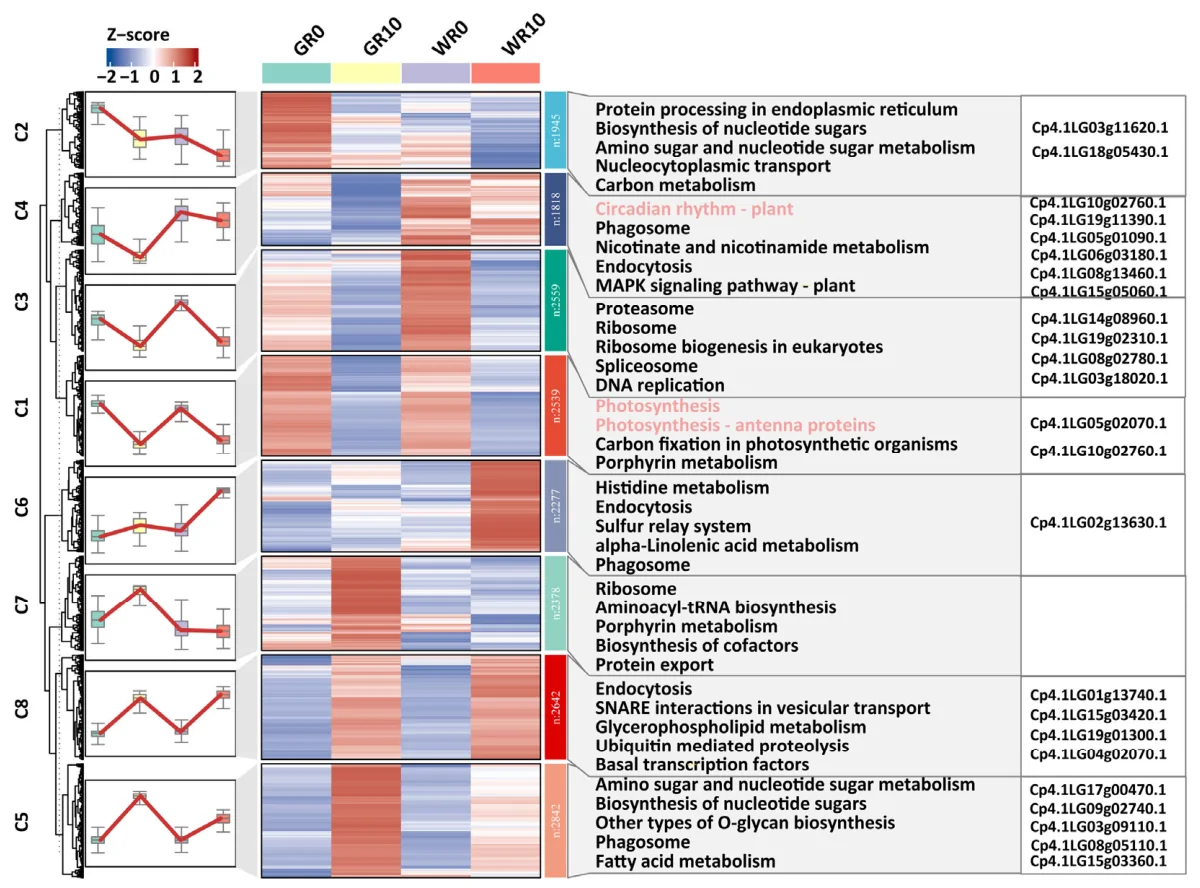
Gene expression heatmap of eight representative co-expression modules. The trendline on the left side of each module represents the gene expression pattern across different sample groups. The number of genes and KEGG enrichment analysis of each module are shown on the right of each module. The top5 KEGG terms are shown. GR = green rind, WR = white rind.

The results showed that the differentially expressed genes could be clustered into eight expression clusters using K-means clustering analysis, with marked expression divergence among different samples. Overall, samples GR0 and WR0 exhibited relatively higher expression levels in clusters 1–4. Specifically, GR0 was mainly enriched in fundamental metabolic processes, including “protein processing in endoplasmic reticulum”, “biosynthesis of nucleotide sugars”, and “carbon metabolism”, whereas WR0 showed higher expression in pathways related to “circadian rhythm-plant”, “MAPK signaling pathway”, and “photosynthesis” pathway. Notably, the significant enrichment of “circadian rhythm-plant” in WR0 suggests enhanced transcriptional activity associated with circadian regulation, indicating a stronger capacity to coordinate endogenous physiological processes with external environmental cues in a temporal manner. Meanwhile, the pronounced enrichment of “photosynthesis-antenna proteins” further indicates that genes involved in light-harvesting complexes were markedly activated in WR0, suggesting a stronger capacity for light absorption and energy capture, as well as a relatively active photosynthetic state at this stage. In contrast, samples GR10 and WR10 displayed overall elevated expression in clusters 5–8. Among them, WR10 exhibited the highest expression in cluster 5, which was mainly associated with “histidine metabolism”, “endocytosis”, and “α-linolenic acid metabolism” pathway. GR10 showed relatively higher expression in clusters 6 and 7, which were primarily enriched in “ribosome”, “aminoacyl-tRNA biosynthesis”, and “vesicular transport pathway”. In contrast, cluster 8 showed higher expression in both GR10 and WR10 than in GR0 and WR0 and was mainly involved in “amino sugar”, “nucleotide sugar metabolism”, and “fatty acid metabolism” pathways. Collectively, these results indicate clear transcriptional differences among samples across different clusters, reflecting a regulatory shift from fundamental metabolism and photosynthesis-related activities in the early stage to membrane transport, metabolic remodeling, and stress response-related processes in the later stage (Figure 6).

### 3.6. Expression of 24 APRR2 Genes in Rind

**Figure 7:**
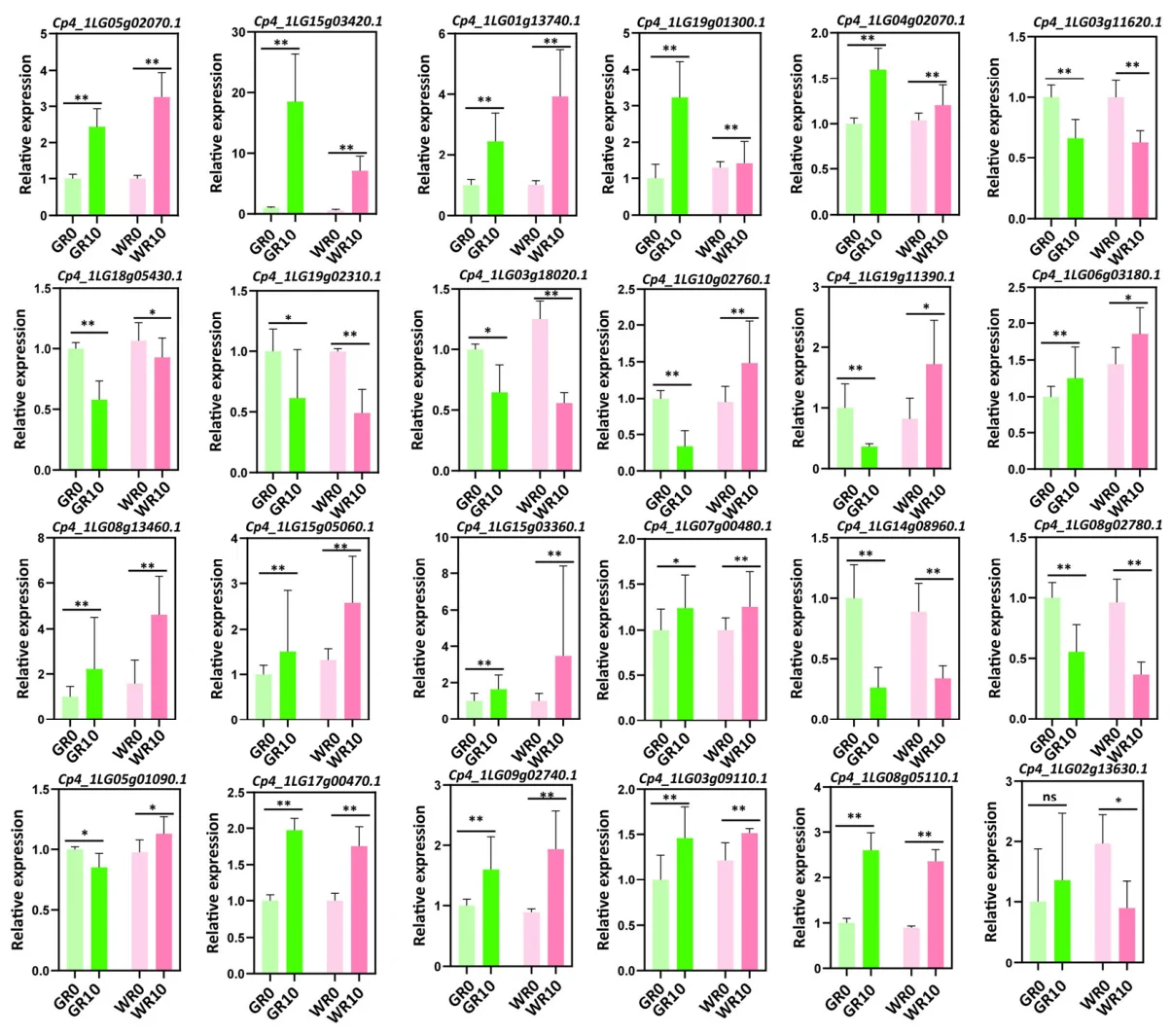
Expression analysis of 24 *APRR2* genes in green rind (GR) and white rind (WR) of *C. pepo*. Expression values (mean ± SE from three biological replicates) were measured using qPCR at 0 and 10 days after pollination (DAP). Significant differences were calculated using the Student’s *t*-test (** *p* ≤ 0.01, * *p* ≤ 0.05) between the indicated time points within each line; inter-color comparisons (GR vs. WR at the same stage) are not annotated in the figure.

To validate the transcriptomic findings, the 24 selected *APRR2* genes were examined using qPCR at 0 and 10 DAP in both rind lines (Figure 7). Based on the direction of expression change at 10 DAP relative to 0 DAP, the 24 genes could be classified into four trend groups (Figure 7). Group I (both GR and WR upregulated, 14 genes) included Cp4.1LG05g02070.1, Cp4.1LG15g03420.1, Cp4.1LG01g13740.1, Cp4.1LG19g01300.1, Cp4.1LG04g02070.1, Cp4.1LG06g03180.1, Cp4.1LG08g13460.1, Cp4.1LG15g05060.1, Cp4.1LG15g03360.1, Cp4.1LG07g00480.1, Cp4.1LG17g00470.1, Cp4.1LG09g02740.1, Cp4.1LG03g09110.1, and Cp4.1LG08g05110.1, which were induced in both lines. Within this group, the magnitude of induction differed. Cp4.1LG15g03420.1, Cp4.1LG19g01300.1, Cp4.1LG04g02070.1, and Cp4.1LG01g13740.1 showed stronger induction in GR. Cp4.1LG08g13460.1, Cp4.1LG15g05060.1, Cp4.1LG15g03360.1, Cp4.1LG06g03180.1, and Cp4.1LG05g02070.1 showed stronger or equal induction in WR. The remaining genes showed comparable induction in both lines. Group II (both GR and WR downregulated, 6 genes) included Cp4.1LG03g11620.1, Cp4.1LG18g05430.1, Cp4.1LG19g02310.1, Cp4.1LG03g18020.1, Cp4.1LG14g08960.1, and Cp4.1LG08g02780.1, which were all downregulated at 10 DAP in both lines, with Cp4.1LG14g08960.1 and Cp4.1LG08g02780.1 being the most strongly repressed. Cp4.1LG03g18020.1 had a higher baseline in WR0 than in GR0, suggesting an early white rind-associated elevation. In Group III (GR upregulated, WR downregulated, 1 gene), Cp4.1LG02g13630.1 was the only gene with opposite trajectories, namely, a non-significant increase in GR but significant decrease in WR. In Group IV (GR downregulated, WR upregulated, 3 genes), Cp4.1LG10g02760.1, Cp4.1LG19g11390.1, and Cp4.1LG05g01090.1 were repressed in GR but induced in WR, representing the clearest WR-associated pattern in the qPCR data. Collectively, the qPCR analysis revealed that the GR/WR differences for the 24 *APRR2* genes are primarily reflected in the direction and magnitude of expression change at 10 DAP, rather than in on/off specificity at any single time point. This trend-based classification provides a more interpretable framework for understanding the regulatory roles of *APRR2* genes in fruit development.

### 3.7. Prediction and Yeast Two-Hybrid Verification of CpAPRR2 Protein Interactions

**Figure 8:**
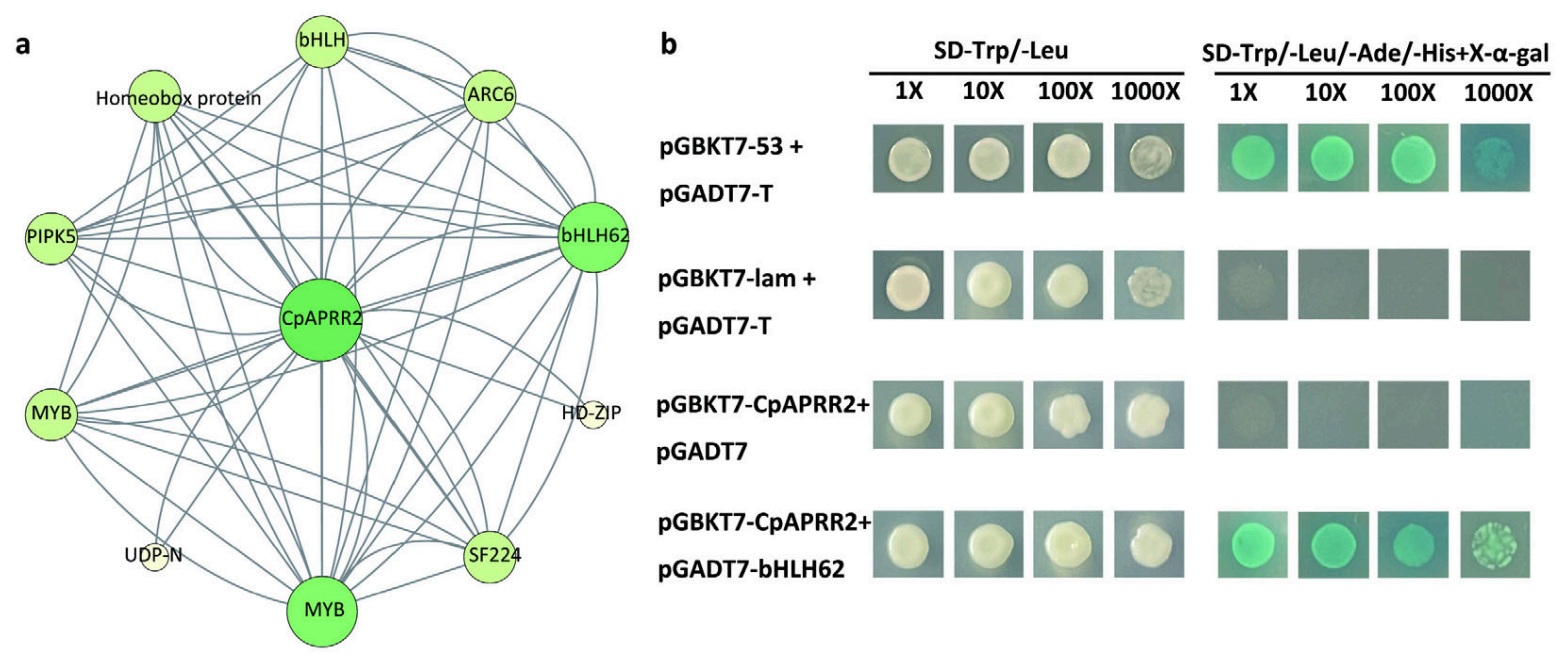
Identification of prey proteins interacting with CpAPRR2. (**a**) Preliminary prediction of interaction protein of CpAPRR2 based on STRING database. (**b**) The protein interaction was measured on the synthetic defined double dropout (SD DDO) medium (left) and SD quadruple dropout (SD QDO) medium (right) (QDO lacks His, Leu, Ade, and Trp; DDO lacks Trp and Leu). BD DNA-binding domains and AD activation domain. pGADT7-Lam and pGBKT7-T was set as the negative control, and pGADT7-53 and pGBKT7-T served as the positive control. BD = DNA-binding domain, AD = activation domain.

To explore the interaction network of the CpAPRR2 protein related to rind color regulation, the “proteins by sequences” function of the STRING database was utilized to predict interacting proteins based on the amino acid sequence of CpAPRR2. The results indicated that CpAPRR2 (Cp4.1LG05g02070.1) exhibited the highest homology (72.39%) with the cucumber homologous gene XP_004148499.1. STRING predicted a total of 10 potential protein interactions for CpAPRR2 (Figure 8a). These 10 candidate protein sequences were further aligned against the *C*. *pepo* genome database, leading to the identification of 2 MYB transcription factors and 2 bHLH transcription factors (Supplementary Table S3). Notably, one of the bHLH genes predicted to interact with CpAPRR2 was identified as a homolog of bHLH62(Cp4.1LG012g10810.1). Subsequently, yeast two-hybrid assays were conducted. The results showed that CpAPRR2 interacted with the bHLH62 protein in yeast (Figure 8b).

## 4. Discussion

In this study, a genome-wide identification of the APRR2 transcription factor family in *C*. *pepo* was conducted. A total of 50 *APRR2* genes were successfully identified, which were unevenly distributed across 20 chromosomes. The number of *APRR2* genes in *C*. *pepo* is significantly higher than that in *A*. *thaliana*, exhibiting a clear lineage-specific expansion. Among the identified genes, Group III accounted for an extremely high proportion, while Group I was completely absent and contained only a single *A*. *thaliana* homolog, reflecting specific selection for subfunctionalization during species differentiation. The large-scale expansion of this family was primarily driven by whole-genome duplication and tandem duplication events on chromosomes Chr05 and Chr15. This pattern is consistent with the general rule of gene family expansion driven by the shared paleotetraploidization event in the Cucurbitales order and aligns with the evolutionary characteristics of other cucurbit species such as cucumber and watermelon [16,24]. Previous studies have shown that cucurbit crops exhibit asymmetric subgenome retention following paleotetraploidization [14]. The lineage-specific amplification of the *APRR2* family has established a novel mechanism for rind color regulation within the Cucurbitaceae family. Furthermore, structural variations (deletions, frameshifts) in *APRR2* genes in species such as cucumber, *C*. *pepo*, and wax gourd directly lead to functional divergence, resulting in fruit color diversity [11,25].

APRR2 homologs across *C*. spp. species universally harbor the REC superfamily core domain, with Motif 1 and Motif 2 exhibiting high conservation and indicating evolutionary conservation within subclades. The *APRR2* gene associated with cucumber rind pigmentation contains a REC domain. Its critical DNA-binding B-motif directly regulates rind color development [26]. Research on the tomato *APRR2* gene confirms that REC domain integrity is essential for pigment regulation [6]. The intact REC domain modulates plastid number and promotes chlorophyll accumulation, whereas domain loss disrupts the pigment biosynthesis pathway. This structural-functional relationship holds true in *C*. spp. and coincides with functional characteristics of SeAPRR2 gene in chayote (*Sechium edule*) [27]. Moreover, the eggplant *SmAPRR2* gene regulates pericarp color by modulating chlorophyll content. SmAPRR2 is highly expressed in green peel cultivars, while a SNP-induced premature stop codon in white peel cultivars results in a truncated REC domain, ultimately causing defective chlorophyll synthesis [28]. Notably, *C*. spp. stem coloration is also regulated by a homologous gene (Cp4.1LG15g03420), where domain deletion correlates directly with reduced chlorophyll content [29]. In the present study, Cp4.1LG05g02070 is identified as a key candidate gene for green rind formation, possessing a complete and intact conserved domain in the genome. Collectively, these findings demonstrate functional conservation among APRR2 and its homologs in pigment regulation, with effective mechanisms strictly dependent on an intact REC structural domain.

Analysis of promoter cis-acting elements revealed that the *APRR2* gene family in *C*. *pepo* is predominantly enriched with four major categories of cis-elements. These include hormone-responsive elements, environmental stress-responsive elements, light-responsive elements, and growth and development regulatory elements. Among these, light-responsive elements were the most abundant. Light, particularly ultraviolet and blue light, is a key signal inducing anthocyanin and carotenoid synthesis. They serve as key signals for inducing the synthesis of chlorophyll and carotenoids, and their function is consistent with that of fruit skin color-related genes such as CsAPRR2 in cucumber and ClAPRR2 in watermelon [11,30]. The promoters of numerous rind color-related genes contain light-responsive elements, demonstrating that *APRR2* genes play a significant role in regulating *C*. *pepo* rind color. This confirms that *C*. *pepo* APRR2 participates in the regulation of pigment synthesis by responding to light signals. The high abundance of both light-responsive and hormone-responsive elements suggests that the *CsAPRR2* gene may contribute to in plant environmental adaptation and hormonal regulation, indicating an association of the CsAPRR2 protein with abiotic stress responses [31,32]. Concurrently, the widespread distribution of hormone-responsive elements, such as those for abscisic acid and auxin, suggests that APRR2 may coordinately regulate fruit development and skin coloration processes through hormonal signaling pathways [33,34]. Furthermore, the absence of low-temperature response elements in certain genes, such as Cp4.1LG02g12250.1 and Cp4.1LG11g07180.1, implies a potentially lower sensitivity to cold environments. Nevertheless, the presence of an element does not necessarily indicate that it is functionally active, and further experimental validation (e.g., GUS reporter assays, ChIP) is required [35].

Based on transcriptomic expression pattern analysis, the *APRR2* gene family in *C*. *pepo* exhibits significant functional differentiation in the regulation of rind color. The DEGs were clustered into eight expression clusters, showing clear expression divergence among different samples, with GR0 and WR0 mainly enriched in clusters 1–4 and GR10 and WR10 in clusters [5,6,7,8]. Notably, WR0 was significantly enriched in circadian rhythm-plant and photosynthesis and antenna proteins, it reveals the conserved role of the *APRR2* gene family genes in the circadian rhythm and photosynthesis of plants, together with relatively active light harvesting and energy capture at the early stage [5]. However, transcriptome analysis was based on bulk RNA-seq of rind tissue, which may mask cell type-specific expression patterns. In addition, genes reflect transcriptional correlations rather than causal relationships. Further studies with spatial transcriptomics and functional validation are needed to confirm these candidates. Furthermore, qPCR validation of the 24 selected APRR2 genes classified them into four trend groups based on the direction of expression change at 10 DAP: 14 genes upregulated in both lines, 6 downregulated in both, 1 upregulated in GR but downregulated in WR, and 3 downregulated in GR but upregulated in WR (Figure 7). Within these groups, the GR/WR differences are reflected primarily in the magnitude and direction of induction rather than in on/off specificity. We interpret these groupings as correlative. Rind color is a complex trait, and the primary determinant of the color difference may not reside in the rind epidermis itself but in subtending tissues (e.g., hypodermis or flesh) or in systemic signals. Because our transcriptomes were derived from rind epidermis at only two developmental time points, these observations establish correlation with rind color, not tissue specificity or causality. A precise assignment will require spatially resolved analyses of isolated tissues combined with a finely resolved temporal (kinetic) analysis of pigment accumulation throughout fruit development. Among them, the core gene Cp4.1LG05g02070.1 was identified as a candidate key factor regulating green rind formation related to photosynthetic pathways in cluster 1. Our previous study was due to a 2227 bp deletion in Cp4.1LG05g02070.1 of the white rind [1].

In this study, our yeast two-hybrid assays demonstrate a physical interaction in yeast between the core *C. pepo* APRR2 and bHLH62, revealing a candidate regulatory interaction that may coordinate rind coloration. This interaction suggests APRR2 may serve as a candidate molecular hub that integrates light signaling and pigment biosynthesis supported by abundant light-responsive cis-elements in APRR2 promoters and transcriptome enrichment of photosynthesis/circadian pathways in early WR0. In *A*. *thaliana*, the bHLH factors may work together with APRR2 to integrate light signals and control the circadian rhythm and photomorphogenesis processes [36,37]. Evolutionarily, APRR2 relies on species-specific partners. Cucumber CsAPRR2 recruits TCPs for chloroplast development, and tomato SlAPRR2 is repressed by KN5-BLH complexes during ripening. However, our findings identify bHLH62 as a candidate co-regulator in *C*. *pepo*, pending in planta validation. This mechanistic divergence highlights APRR2’s adaptable role as a pigment biosynthesis scaffold, where impaired complex assembly underlies white rind phenotypes [10,11,12,13]. Nevertheless, because the interaction was detected in a heterologous yeast system, it remains a candidate interaction. Specifically, establishing it in planta will require nuclear co-localization of CpAPRR2 and CpbHLH62 at all stages of rind development, complemented by independent assays such as BiFC or Co-IP. In summary, the APRR2 family exhibits profound functional divergence during rind development in *C*. *pepo*. Their distinct spatiotemporal expression patterns are closely linked to rind color formation and developmental progression. This analysis provides critical evidence for identifying key genes regulating rind color and elucidating their molecular mechanisms.

## Figures and Tables

**Table 1:** The physical and chemical properties of *APRR2* family proteins in *C. pepo.*

Gene ID	Number of Amino Acid	Molecular Weight	pI	Instability Index	Aliphatic Index	Grand Average of Hydropathicity	Subcellular Localization
Cp4.1LG05g02070.1	538	60,328.25	5.46	43.62	72.29	−0.565	nucleus
Cp4.1LG05g02060.1	492	55,019.57	4.96	45.51	70.33	−0.595	nucleus
Cp4.1LG15g03420.1	523	58,811.81	6.32	46.35	73.25	−0.551	nucleus
Cp4.1LG16g04170.1	436	48,380.32	6.2	60.42	67.06	−0.71	nucleus
Cp4.1LG17g00470.1	683	75,489.63	5.47	50.19	75.05	−0.488	nucleus
Cp4.1LG03g11620.1	692	77,092.34	8.03	51.71	77.99	−0.464	nucleus
Cp4.1LG14g08960.1	542	60,538.14	5.41	52.16	75.74	−0.594	nucleus
Cp4.1LG06g03180.1	570	63,989.3	6.58	36.79	78.49	−0.581	nucleus
Cp4.1LG08g05110.1	627	69,007.66	6.12	52.86	72.15	−0.523	nucleus
Cp4.1LG18g05430.1	517	57,296.81	5.55	40.71	85.94	−0.398	nucleus
Cp4.1LG15g03360.1	442	49,536.08	6.62	44.73	68.8	−0.597	nucleus
Cp4.1LG05g14170.1	394	43,475.98	6.15	51.25	71.07	−0.604	nucleus
Cp4.1LG07g00480.1	571	64,509.08	5.32	42.58	76.92	−0.444	nucleus
Cp4.1LG17g02770.1	416	46,728.41	9.25	39.69	65.1	−0.61	nucleus
Cp4.1LGO9g01140.1	455	50,841.95	8.99	35.14	80.95	−0.439	nucleus
Cp4.1LG18g04520.1	428	47,126.31	6.3	57.65	63.88	−0.785	nucleus
Cp4.1LG10g02760.1	201	21,584.1	9.44	42.84	66.42	−0.79	nucleus
Cp4.1LG19g11390.1	377	42,045.18	5.99	50.45	73.47	−0.744	nucleus
Cp4.1LG10g10650.1	303	32,562.47	6.42	59.12	71.55	−0.462	nucleus
Cp4.1LG08g13460.1	288	31,126.69	6.36	48.02	69.1	−0.525	nucleus
Cp4.1LG02g13630.1	368	41,162.34	8.09	41.23	61.2	−0.582	nucleus
Cp4.1LG16z06120.1	288	32,000.91	5.94	46.09	64.72	−0.613	nucleus
Cp4.1LG03g04120.1	552	59,343.42	6.26	53.75	70.62	−0.451	nucleus
Cp4.1LG14g05820.1	423	47,766.67	9.05	49.88	76.26	−0.587	nucleus
Cp4.1LG01g17980.1	362	39,934.51	7.16	59.51	63.9	−0.664	nucleus
Cp4.1LG07g09900.1	484	52,425.14	6.58	55.04	68.22	−0.747	nucleus
Cp4.1LG01g13740.1	351	38,574.95	7.77	69.45	65.24	−0.733	nucleus
Cp4.1LG04g08210.1	370	40,732.76	8.25	65.7	64.11	−0.699	nucleus
Cp4.1LG15g05060.1	344	38,326.16	7.51	60.76	68.58	−0.7	nucleus
Cp4.1LG10g12360.1	386	43,378.41	6.33	60.16	59.66	−0.664	nucleus
Cp4.1LG05g08380.1	441	48,889.92	5.31	54.82	63.51	−0.868	nucleus
Cp4.1LG11g00530.1	489	55,656.07	5.26	49.02	79.1	−0.471	nucleus
Cp4.1LG04g09020.1	688	75,099.41	6	36.76	80.16	−0.459	nucleus
Cp4.1LG06g05660.1	228	25,981.5	8.96	77.4	72.63	−0.673	nucleus
Cp4.1LG13g06810.1	378	41,833.87	6.87	65.1	64.29	−0.67	nucleus
Cp4. 1LG03g09110.1	216	24,944.04	8.27	53.03	63.66	−0.969	nucleus
Cp4.1LG04g02070.1	442	49,255.45	5.1	51.97	67.08	−0.765	nucleus
Cp4.1LG19g01300.1	378	42,757.2	8.43	61.45	60.9	−0.64	nucleus
Cp4.1LG19g02310.1	299	32,203.26	6.36	57.8	72.81	−0.429	nucleus
Cp4.1LG09g02740.1	344	37,971.08	8.91	61.77	68.52	−0.647	nucleus
Cp4.1LG05g01090.1	489	54,327.31	5.08	47.09	72.94	−0.638	nucleus
Cp4.1LG02g02600.1	231	26,521.1	8.63	78.88	73.38	−0.711	nucleus
Cp4.1LG03g18020.1	314	33,937.59	6.62	55.05	74.33	−0.409	nucleus
Cp4.1LG08g02780.1	383	42,074.47	8.17	56.82	83.08	−0.347	nucleus
Cp4.1LG12g03000.1	671	74,607.03	5.83	45.02	75.05	−0.469	nucleus
Cp4.1LG03g09290.1	383	41,855.77	7.73	32.78	68.77	−0.711	nucleus
Cp4.1LG02g12250.1	221	25,481.1	8.91	42.02	76.79	−0.823	nucleus
Cp4.1LG05g12030.1	385	43,110.45	8.89	61.59	66.55	−0.737	nucleus
Cp4.1LG11g07180.1	249	28,021.72	6.42	48.32	75.18	−0.69	nucleus
Cp4.1LG20g08290.1	332	36,419.69	5.91	37.59	58.43	−0.674	nucleus

## Data Availability

The data that support the findings of this study are available from the corresponding author upon reasonable request.

## Supplementary Materials

The following supporting information can be downloaded at: https://www.mdpi.com/article/10.3390/genes17091063/s1. Table S1: Primer sequences of genes linked to Yeast two hybrid vector; Table S2. qRT-PCR primers; Table S3: Information about predictive interacting proteins.
