# Supplementary material for: Genome-Wide Identification of the APRR2 Gene Family and Rind Color Trait Analysis in Zucchini (Cucurbita pepo)
Source: Genes (Basel). 2026 Sep 1;17(9):1063. doi: 10.3390/genes17091063 (PMC13606185; doi:10.3390/genes17091063)
Supplement: Supplementary file 1 [file genes-17-01063-s001.zip › genes-4521339-supplementary.pdf]

Supplementary Table S1 Primer sequences of genes linked to Yeast two hybrid vector

Primer name	Forward primer sequence (5'-3')	Reverse primer sequence (5'-3')
AD-CpAPRR2	CCCGGGTGGGCATCGATACGATG GTTTGCACTGCCGACG	CATCTGCAGCTCGAGCTCGATGG GAGGTTTGGAGCCGTTG
BD- CpAPRR2	CATGGAGGCCGAATTCCCGGATG GTTTGCACTGCCGACG	CGGCCGCTGCAGGTCGACGGGA GGTTTGGAGCCGTTG
AD- BHLH62	CCCGGGTGGGCATCGATACGATG GAGAACGAATTCTTCC	CATCTGCAGCTCGAGCTCGATTC ATAGTTCAATTTTCATTTGC
BD- BHLH62	CATGGAGGCCGAATTCCCGGATG GAGAACGAATTCTTCC	CGGCCGCTGCAGGTCGACTCATA GTTCAATTTTCATTTGC

Supplementary Table S2 QRT-PCR primers

GeneID	Forward primer	Reverse primer
Cpaction	CCTCTCAATCCCAAAGCTAAC	CGGCCTGGATAGCAACATAC
Cp4.1LG05g02070.1	TGGCGTCAAAAGTGGTCCTT	GGCTCCAGTCCACCTTTGTT
Cp4.1LG15g03420.1	CCTGCACCGATGAGAAGGAA	TGGCAAGTCCATGGAAGCTC
Cp4.1LG01g13740.1	CAGCCGTTTCAGAAGGGGAA	CACCGCCTTTGTTTTCTCCG
Cp4.1LG19g01300.1	GTGCTTCTGTGGGCTATGGA	AATTGCTTTTGGCGTTGCCT
Cp4.1LG04g02070.1	TGCTCCACAGAACCAAACGA	CCGCCAAGTTTATCGACAGC
Cp4.1LG03g11620.1	TCTTCGAACTCTCGCTGCTG	TAGGCATGCTCGATTTCGCT
Cp4.1LG18g05430.1	TGATGAAGGGGATAGCGCAC	TTTGCCTTCGGTTCGAGCTT
Cp4.1LG19g02310.1	GGGTTTATCCAGTGGGGGTG	ACGAGCGGAGTGAAGCAAAT
Cp4.1LG03g18020.1	CGACGGAGAACTCGAAGGAC	ATCTGTGCTCGTAACGCCTC
Cp4.1LG10g02760.1	TCAAGTGGCAACGCCAAAAC	TCGTGTTCTCCCGGTATCCA
Cp4.1LG06g03180.1	TTGAGCAGCGGAGATCCTTC	TTCAATCGACCGAGCATCCC
Cp4.1LG19g11390.1	ACCCCAATCTCCGATCCCTT	TGGTCTGTTGGTGTTGGGAC
Cp4.1LG08g13460.1	CACCCGTTTCAGATCCTGCT	CAAACGGCAGGAAATGGTCG
Cp4.1LG15g05060.1	GGTCGAATCTACGGACGGTG	TGCGACTTGTGAACCTCCAA
Cp4.1LG15g03360.1	ATTCGTATGCAGGAGCAGGG	GAGGCATGCCGTAGCTCTTA
Cp4.1LG07g00480.1	ATCAGCGACGTAAACATGCC	CACAAGCCCCATGTTGAACC
Cp4.1LG14g08960.1	CGCTTGGTGAAGGCATTTCC	CGTCCCACTCGAATTACCGA
Cp4.1LG08g02780.1	ACTCGGGAAGCAATCGTTCA	CACTCGTAAAGCCTCCGTGA
Cp4.1LG05g01090.1	GCAAGTGCAGCAGTCTCAAA	AAGTTCTGGTGTCCATCGCA
Cp4.1LG17g00470.1	GTTGCCAGTCACCTCCAGAA	GAGATGGCCAGTGACCGAAA
Cp4.1LG09g02740.1	TCCATCTGAAACAGAGGCGG	GGTGTAACTCCGACGACCAG
Cp4.1LG03g09110.1	AAGCCACACCAAAGCAACTC	TTGTCTCGCTGCTTCCACC
Cp4.1LG08g05110.1	TCGAGAAGGCTGTTCCGAAG	TTCAACGTCGAGCCAAATGC
Cp4.1LG02g13630.1	CAGGGATCTTCCAACGAGGG	AGGGCATCGGAGTAGGAACT

Supplementary Table S3 Information about predictive interacting proteins

Number	Gene ID in cucumber	Functional annotation	Gene ID in *C. pepo*	Functional annotation	Identity
1	*XP_004148499.1*	Two-component response regulator-like protein APRR2	*Cp4.1LG05g02070*	Two-component response regulator-like protein APRR2	72.39%
2	*XP_004140812.1*	Uncharacterized protein LOC101204775	*Cp4.1LG14g04420*	Homeobox protein (HD-ZIP)	74.53%
3	*XP_004141447.1*	Myb-related protein 306-like; Upstream in-frame stop codon	*Cp4.1LG01g22850*	R2R3-MYB transcription factor	71.62%
4	*XP_004170982.1*	Myb-related protein 306-like			
5	*XP_004159616.1*	Transcription factor myb76-like; Uncharacterized protein; Upstream in-frame stop codon	*Cp4.1LG14g01290*	MYB-related transcription factor	76.67%
6	*XP_004166810.1*	Protein ACCUMULATION AND REPLICATION OF CHLOROPLASTS 3-like	*Cp4.1LG13g06310*	1-phosphatidylinositol-4-phosphate 5-kinase, putative PIPK5	84.97%
7	*XP_004152763.1*	Protein ACCUMULATION AND REPLICATION OF CHLOROPLASTS 6, chloroplastic-like	*Cp4.1LG11g02290*	ARC6	85.97%
8	*XP_004169691.1*	Transcription factor bhlh78-like; Uncharacterized protein; Upstream in-frame stop codon	*Cp4.1LG03g12280*	BHLH transcription factor	81.82%
9	*XP_004170614.1*	Uncharacterized protein; Transcription factor bHLH62-like	*Cp4.1LG12g10810*	BHLH transcription factor	79.14%
10	*XP_004136854.1*	Dynamin-like protein arc5 isoform x1; Belongs to the TRAFAC class	*Cp4.1LG04g06880*	Dynamin family protein, putative, expressed	93.77%

		dynamin-like GTPase superfamily			
11	*XP_004163623.1*	Uncharacterized protein loc101208336; Uncharacterized LOC101208336	*Cp4.1LG05g11160*	UDP-N-acetylmuramoyl-L-alanyl-D-glutamate--2, 6-diaminopimelate ligase	80.9%
